# Examining the Importance of Green Food in the Restaurant Industry: Focusing on Behavioral Intentions to Eat Insects

**DOI:** 10.3390/ijerph18041905

**Published:** 2021-02-16

**Authors:** Jinsoo Hwang, Hyunjoon Kim

**Affiliations:** 1 Department of Food Service, The College of Hospitality and Tourism Management, Sejong University, Seoul 143-747, Korea; jhwang@sejong.ac.kr; 2 The Department of Tourism Management, The College of Business Administration, Dong-A University, Busan 49236, Korea

**Keywords:** edible insect restaurants (EIRs), environmentally friendly, psychological benefits (PBs), attitude, behavioral intentions

## Abstract

This study analyzed the psychological benefits of environmentally friendly edible insect restaurants, by proposing that three subdimensions of psychological benefits positively affect attitude. Attitude was hypothesized to play an important role in the formation of desire and two subdimensions of behavioral intentions: intentions to use and willingness to pay more. A research model was verified using responses from 419 respondents collected in Korea. Data analysis indicated that (1) warm glow, (2) self-expressive benefits, and (3) nature experiences form attitude and that attitude helps to increase desire, which in turn positively enhances behavioral intentions. The data analysis results supported the importance of the psychological benefits of environmentally friendly edible insect restaurants.

## 1. Introduction

Globally, the damage caused by environmental pollution is serious. Environmental pollution results in global warming, which is when the average temperature of the Earth rises rapidly due to greenhouse gases in the atmosphere [1]. In particular, global warming is a major cause of abnormal climate changes, such as droughts, floods, and desertification [2,3]. For this reason, consumers have become more aware of environmental pollution, which leads to changes in their consumption behaviors [4,5]. For example, consumers want to buy ecofriendly products to protect our environment, and they are even willing to pay more for such products [6,7]. In order to meet consumers’ environmental needs, companies are also making many efforts to implement environmental management [8,9].

Recently, environmental protection has become an important issue in the restaurant industry, and many scholars and practitioners are paying attention to the environmentally friendly role of edible insect restaurants (hereafter EIRs). For example, edible insects aid recycling of animal waste and further reduce greenhouse gas emissions [10,11]. In addition, many restaurant managers and chefs are working hard to develop new menus using edible insects to create new customers [12]. The report “Edible Insects: Future Prospects for Food and Feed Security” released by the Food and Agriculture Organization of the United Nation in 2013 also indicated that edible insects (hereafter EI) are sustainable foodstuff for the future. As such, people are getting more and more interested an EI in the food service industry, but there is a lack of academic research.

More importantly, environmentally friendly services, such as EIRs, have a vital role to play in giving psychological benefits (hereafter PBs) to consumers [13,14]. PBs are feelings of trust in a certain thing or person that leads to peace of mind [15]. PBs have great meaning in green research because consumers are comfortable in thinking that they participate in the environmental movement by purchasing green products/services [16]. In the same vein, consumers are more likely to be mentally comforted that they can protect the environment by dining at environmentally friendly EIRs. For this reason, it is meaningful to examine the significant role of PBs in the field of EIRs.

In summary, this paper was designed to apply the concept of PBs to the context of EIRs using structural equation modeling (SEM) analysis for the first time. Specifically, the current paper examined (1) the effect of three subdimensions of PBs, namely, warm glow (hereafter WG), self-expressive benefits (hereafter SEBs), and nature experiences (NEs) on attitude, (2) the important role of attitude in the formation of desire and intentions to use and willingness to pay more, and (3) the influence of desire on intentions to use and willingness to pay more. The results of this study will provide important information for managers who currently operate or prepare EIRs to understand the importance of the ecofriendly role of EIRs and to establish marketing tactics to improve consumer behavioral intentions. In addition, the study, as shown below, consists of six sections: (1) Introduction, (2) Literature review, (3) Methodology, (4) Discussion and implications, (5) Conclusion, and (6) Limitations and future research.

## 2. Literature Review

### 2.1. Edible Insect Restaurants and Their Ecofriendly Role

According to Hwang and Choe [17], EIRs refer to “a commercial property serving customers a specialty cuisine consisting of EIs” (p. 1). Globally, the number of EIRs is on the rise [18]. For instance, among the diverse menus of EIRs in Thailand, insect salads made from eggs of water bugs and ants. In addition, tom yam soups based on silkworm pupae are popular [19]. In China, EI foods made from bamboo insects, grasshoppers, silkworm pupae, and stink bugs are loved by the Chinese [20]. In addition, The Fonda Don Bon Restaurant in Mexico City is popular, with EI meals made from ant larvae, grasshoppers, and maggy worms, which are sold at about 25$ US [21].

The popularity of EIRs results from many of the advantages of eating EIs. More than two billion people in the world are estimated to consume insects as a regular part of their diet [18], and the estimated global market volume of EI will be US$8 billion by 2030 [22]. Approximately 1900 species have been identified as EIs across different continents [23]. Crickets, mealworms, buffalo worms, grasshoppers, ants, silkworms, and cicadas are examples of commonly consumed insects by people [18]. Crickets are loved as they provide excellent crunchy flavor and are rich in protein and amino acids that easily break down in the human digestive system. Grasshoppers are particularly well known as chapulines in Mexican cuisine, and are simmered in mole sauce or tossed into tacos. Calories from EIs run to 776.9 kcal per 100 g which is superior to that from soy, and the composition of six fatty acids and unsaturated omega-3 in mealworms is higher than that in beef or pork [24].

More importantly, the environmental aspects of EIs have consistently attracted great attention (e.g., [18,25,26]). The livestock production systems are known to adversely affect the environment [17]. Extensive land is needed to raise livestock, which leads to deforestation and desertification [27]. Furthermore, livestock emit greenhouse gas emissions that have a serious impact on climate change. Such climate change brings great problems to human food production [28]. On the other hand, EIs require far less feed than livestock, and the ammonia emissions of EIs are also very low [29]. Global warming potential levels are also the lowest in EIs compared to animals like beef and chicken [30]. 

In summary, livestock breeding has a major impact on the destruction of the global environment, such as global warming and desertification. Therefore, EIs are gaining attention as an alternative source of food, but there is a lack of research on EIs in the restaurant industry. 

### 2.2. Psychological Benefits

In consumer research, PBs refer to the mental stability that consumers perceive after purchasing a particular product/service [31]. In recent years, the concept of PBs has been widely used in green research explaining environmentally friendly behavior as consumers are concerned about environmental protection [16]. In other words, consumers want to use ecofriendly products or services because they are well aware that their ecofriendly consumption helps to protect the environment [14]. Prior studies have commonly suggested the following three subdimensions of PBs in green research: (1) WG, (2) SEBs, and (3) NEs (e.g., [13,14,16,32,33]). 

The first dimension of PBs is WG, which is defined as “satisfaction that goes beyond the benefits derived from aggregate provision of a public good through proenvironmental behavior” [34] (p. 239). WG is a moral satisfaction with what has been done to protect the environment, so it is known as self-satisfaction with the moral responsibility for the actions taken to protect the environment [35,36]. Similarly, Harbaugh, Mayr, and Burghart [37] suggested that WG is a reward that humans gain from helping others. For this reason, WG is called ‘intrinsic satisfaction’ [38]. In addition, Brekke, Kverndokk, and Nyborg [39] examined the relationship between social responsibility and WG, and they found that the higher the level of the social responsibility, the higher WG.

The second dimension of PBs is SEBs. The concept of SEBs refers to the benefits consumers derive from their efforts to express concerns about natural environment problems to others [32,40]. SEBs are theoretically supported by signaling theory [16,41,42], suggesting that humans tend to express their likes and dislikes for any phenomenon or thing based on their beliefs. Green consumer behavior can be explained by self-expressive benefit. Green consumers are interested in protecting the environment, so they want to buy green products to express it, which consequently leads to high levels of SEBs [43]. Hu [44] also argued that people have a higher level of SEBs when they express interest in protecting the natural environment.

The third dimension of PBs is NEs. Nature means a lot in our lives. In philosophy, nature is the foundation of our lives [45], so they are inextricably linked. In particular, our mental health is greatly helped by contact with nature, such as mountains, rivers and lakes, which enhances the well-being perception [46,47]. NEs serve to stimulate ecofriendly consumer purchases [14,48]. For example, when a company promotes products to consumers with higher levels of NEs through ads that emphasize the natural environment, the advertising effect is greater than those with lower levels of NEs.

### 2.3. Effect of Psychological Benefits on Attitude

This study firstly hypothesized the effect of PBs of EIRs on attitude towards using EIRs based on the following the rationale. Attitude refers to “the degree to which a person has a favorable or unfavorable evaluation or appraisal of the behavior” [49] (p. 188). Many prior studies have long examined the important role of attitude in consumer research, and they found that it is a significant factor influencing individuals’ decision making process for purchase (e.g., [50,51,52]). 

It is widely accepted that customers have a positive attitude toward using a certain product when they are comforted with the use of the product that can show their proenvironmental beliefs [13], suggesting that PBs are a critical factor affecting attitude. Furthermore, empirical research has also shown that PBs are an important predictor of attitude. For instance, Hu [44] argued that when consumers receive PBs from a certain product, they have positive feelings about using it. In addition, Hwang and Choi [32] showed that when customers receive high levels of PBs, including WG, SEBs, and NEs from green airline brands, they tend to have a positive image of the brands. More recently, Hwang et al. [16] also showed that if consumers have high levels of WG, SEBs, and NEs when they will have a good feeling. Based on this discussion, this study proposed the following hypotheses.

**Hypothesis** **1** **(H1).** *Warm glow positively affects attitude.*

**Hypothesis** **2** **(H2).** *Self-expressive benefits positively affect attitude.*

**Hypothesis** **3** **(H3).** *Nature experiences positively affect attitude.*

### 2.4. Effect of Attitude on Desire and Behavioral Intentions

Since desire was introduced by the model of goal-directed behavior (also known as MGB), it has been applied in various fields, including bicycle travel, casino, cruise, and festival, [53,54,55,56]. Desire refers to “a state of mind whereby an agent has a personal motivation to perform an action or to achieve a goal” [57] (p. 71). People’s desire is a state in which they crave specific actions which are mainly formed through internal stimuli such as past experiences [57,58]. For instance, if consumers have a good experience with any product, they will have high a level of desire to use the product.

According to MGB, consumers are more likely to desire to use a certain product if they have a positive attitude towards the product [58], suggesting that attitude is a significant predictor of desire. Previous research has also supported the relationship between two concepts. For example, Han, Kim, and Lee [6] examined the relationship between attitude and desire in the context of an environmentally responsible museum. They argued that attitude is a significant factor affecting desire. More recently, Hwang and Lyu [59] investigated the influence of attitude on desire in the airline industry, and they found that when passengers have a favorable attitude towards using an environmentally friendly airline, they are more likely to have high levels of desire to use the airline. Thus, this study proposed the following hypothesis.

**Hypothesis** **4** **(H4).** *Attitude has a positive influence on desire.*

In addition, the current study proposed the influence of attitude on behavioral intentions. According to Oliver [60], behavioral intentions refer to ‘‘a stated likelihood to engage in a behavior” (p. 28). It is widely accepted that behavioral intentions have the following two subdimensions: intentions to use and willingness to pay more [61,62,63]. Intentions to use can be defined as “the degree to which a person has formulated conscious plans to perform or not perform some specified future behavior” [64] (p. 214). Since intentions to use are created according to the evaluation after using a product, people with high levels of intentions to use tend to show actual consumption [65]. Second, willingness to pay more can be defined as the amount people are willing to pay for their preferred brand over other comparable brands [66,67]. Willingness to pay more has a great meaning in ecofriendly management because consumers want to buy ecofriendly products to protect the environment, despite being more expensive than general products [68,69].

There are many theoretical bases, such as the theories of planned behavior, of reasoned action, and of repurchase decision-making, for the relationship between attitude and behavioral intentions [50,70,71]. These theories suggested that consumers’ behavioral intentions toward a product depend on the attitude that the consumers have toward the product. Many studies have also found the relationship between attitude and behavioral intentions. For instance, Han and Hyun [72] developed a theoretical model to identify the effect of attitude on behavioral intentions in the field of an environmentally responsible museum. They found that attitude plays a critical role in creating behavioral intentions. In addition, Kim and Hwang [73] explored the relationship between attitude and behavioral intention among 401 potential consumers in the context of food deliver services using drones. They suggested that attitude positively affects behavioral intentions.

**Hypothesis** **5** **(H5).** *Attitude positively affects intentions to use.*

**Hypothesis** **6** **(H6).** *Attitude positively affects willingness to pay more.*

### 2.5. Effect of Desire on Behavioral Intentions

The relationship between desire and behavioral intention is theoretically supported by the MGB [74], suggesting that if people have a high level of desire to engage in a particular action, they are more likely to take the action. The attention, interest, desire, and actions model also theoretically supported the relationship between desire and behavioral intentions [75], indicating that desire is a significant antecedent of behavioral intentions. Prior research has shown the effect of desire on behavioral intentions. For instance, Meng and Han [56] investigated the role of desire in forming behavioral intentions using 394 bicycle tourists. They showed that when tourists desire to take a bicycle trip, they have high levels of behavioral intention. Lee et al. [76] also investigated the relationship between desire and behavioral intentions using 529 tourists in the context of pop-culture-featured destinations. They suggested that desire is a critical factor influencing behavioral intentions.

**Hypothesis** **7** **(H7).** *Desire positively affects intentions to use.*

**Hypothesis** **8** **(H8).** *Desire positively affects willingness to pay more.*

### 2.6. Proposed Model

**Figure 1:**
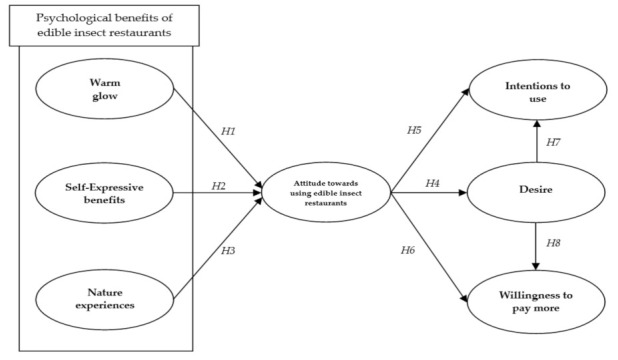
The proposed conceptual model.

Based on these eight hypotheses, the research model is suggested (see Figure 1).

## 3. Methodology

### 3.1. Measurement Items

Measurements of seven constructs were adapted from previous studies and revised to fit the context of ecofriendly EIRs. First, PBs consisted of three subdimensions, namely WG, SEBs, and NEs, which were measured with nine items employed by Hartmann and Apaolaza-Ibáñez [14], Hwang and Choi [32], and Hwang et al. [16]. Second, the concept of attitude was measured based on the three items cited from Ajzen [49] and Han and Hyun [72]. Third, behavioral intentions comprised two subdimensions, namely (1) intentions to use and (2) willingness to pay more, which were measured with six items adapted from Hwang and Hyun [52] and Zeithaml, Berry, and Parasuraman [77]. 

In addition, a seven-point Likert-type, ranging from 1 (strongly disagree) to 7 (strongly agree) was used to measure the seven constructs. In order to identify content validity, three expert groups were invited to review the initial questionnaire. The three expert groups consisted of professors majoring in restaurant management, master’s and PhD students with experience working in the restaurant industry, and managers currently working in the restaurant industry. The review of the expert groups confirmed that the content validity of the questionnaire was not a problem.

### 3.2. Data Collection

Since there are not many EIRs in Korea, the respondents did not have a good understanding of such restaurants, so we showed two newspaper articles and one video explaining the environmental role of EIRs to the respondents. The study conducted a pretest based on 30 restaurant patrons using an online survey, and the results showed that the Cronbach alpha values of all measurement items exceeded 0.7, ensuring the reliability of the measurements [78]. For the main data collection, we used the ‘E’ company, which is one of the largest online survey companies in Korea. In the same way as the pretest, we showed respondents two newspaper articles and one video before starting the survey. The company sent invitation emails to 6479 panels, of whom 450 completed the survey. However, 31 outliers were deleted. As a result, statistical analysis was performed based on 419 responses. In order to test proposed hypotheses, this study used confirmatory factor and structural equation modeling analyses based on the Analysis of Moment Structures (AMOS) program.

### 3.3. Profile of Respondents

Table 1 provides the profile of the respondents. The 419 respondents consisted of 211 males (50.4%) and 208 females (49.6%). The mean age of respondents was 37.95 years. With regard to monthly household income, 122 respondents (29.1%) answered that their income was between 1001$ US and 2000$ US. In addition, about half of respondents (*n* = 218, 52%) were married. Lastly, in terms of education level, 54.4% (*n* = 228) held a bachelor’s degree.

### 3.4. Confirmatory Factor Analysis (CFA)

Table 2 shows the results of CFA. The results indicated that the proposed model had an acceptable fit to the data including normed fit index (NFI), comparative fit index (CFI), Tucker–Lewis index (TLI), and root mean square error of approximation (RMSEA) [79]. The values of all the factor loadings were higher than 0.871 and were all significant (*p* > 0.001).

As shown in Table 3, all of the composite reliability values were greater than the minimum threshold of 0.70 [80], which suggested that the measurement items had high levels of internal consistency. In addition, convergent validity was verified as all of the average variance extracted (AVE) values exceeded 0.50 [81]. Lastly, the AVE values were greater than the squared correlations between constructs, which indicated high levels of discriminant validity [80].

### 3.5. Structural Equation Modeling (SEM)

**Figure 2:**
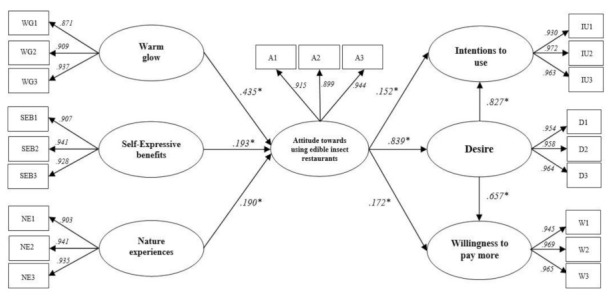
Standardized theoretical path coefficients. Note: * *p* < 0.05

SEM analysis was conducted in order to identify the eight hypotheses. The structural model had an appropriate fit to the data [82]. The SEM analysis results revealed that all hypotheses were statistically supported at *p* < 0.05. More specifically, the results indicated that WG (β = 0.435, *p* < 0.05), SEBs (β = 0.193, *p* < 0.05), and NEs (β = 0.190, *p* < 0.05) positively affected attitude, which supports hypotheses 1, 2, and 3. In addition, attitude plays an important role in the formation of desire (β = 839, *p* < 0.05), intentions to use (β = 152, *p* < 0.05), and willingness to pay more (β = 172, *p* < 0.05). Hence, hypotheses 4, 5 and 6 were supported. Lastly, desire aided to increase intentions to use (β = 827, *p* < 0.05), and willingness to pay more (β = 657, *p* < 0.05). Thus, hypotheses 7 and 8 were supported. The results of SEM analysis are summarized in Table 4 and Figure 2.

## 4. Discussion and Implications

### 4.1. Theoretical Implications

First, this study showed that WG is an important predictor of attitude, which suggested that consumers are more likely to have a positive attitude towards visiting EIRs when they feel that eating at the restaurants contributes to the well-being of humanity and nature. This finding indicated the importance of WG in green consumer behavior, which has been confirmed by previous studies in diverse fields [16,32,40]. They indicated that WG forms positive emotions or images about an object. For instance, Hwang and Choi [32] found that when passengers perceive high levels of WG, they tend to have a positive image. Unlike prior studies, the current study identified that WG is a vital factor in forming attitude in the context of EIRs for the first time, which is a critical theoretical implication of this paper.

Second, the current paper confirmed that SEBs are a crucial factor influencing attitude. The finding indicates that when people customers feel that they can show their concerns about the environment through visiting EIRs, they have a favorable attitude towards using the restaurants. The results of this paper are partially similar to prior studies in green research [16,32,83], which suggested that SEBs are a significant predictor of attitude, emotion, and image. For instance, Ahmad and Thyagaraj [83] suggested that when customers have a high level of SEBs through a particular green brand, they tend to have a favorable attitude towards using the brand. Compared with prior research, the theoretical implication of this study is that it extended the concept of SEBs to the field of EIRs and found a new outcome variable of SEBs.

Third, the data analysis revealed that there is a positive relationship between NEs and attitude. In other words, if consumers perceive that EIRs make them feel close to natural environment including forests and mountains, they will have a positive attitude towards using the restaurants. Prior studies have also verified the relationship (e.g., [14,16,32]). For example, Hartmann and Apaolaza-Ibáñez [14] showed that when a certain green energy brand is reminiscent of being with NEs, consumers have a favorable attitude towards using the brand. In this respect, the current paper replicated and also further expanded the existing literature by finding the significance of NEs in the context of EIRs.

Fourth, another significant theoretical implication of this paper was to determine the role of attitude in the formation of three outcomes in the field of EIRs for the first time. To be specific, the SEM results indicated that attitude positively affects desire, which in turn positively affects intentions to use, and willingness to pay more. That is, when consumers have a favorable attitude towards visiting EIRs, they would desire to visit them and further to have high levels of intentions to use, and willingness to pay more. These findings are in line with existing studies (e.g., [59,72,76]), which indicated that attitude is an important factor influencing desire, intentions to use, and willingness to pay more. These findings have theoretical implications in that attitude was first introduced into the field of EIRs, thereby extending its applicability.

### 4.2. Managerial Implications

First, the data analysis result showed the effect of WG on attitude. This finding suggests the following important managerial implications. First and foremost, in order to make consumers’ attitudes positive about visiting EIRs, it should be emphasized that the restaurants play an important role in protecting the environment. EIRs can contribute strongly to environmental protection in various aspects, such as greenhouse gas emissions and global warming [28,30]. More importantly, consumers are willing to purchase ecofriendly products to satisfy their environmental needs, despite costing more [84,85]. Therefore, restaurant companies operating or preparing EIRs need to explain the negative impacts of livestock on the environment. In addition, if consumers are well informed about the ecofriendly role of EIRs, they will have a good attitude towards using the restaurants. Especially in countries where there are few EIRs, such as Korea, consumers do not know the ecofriendly role of EIRs well, so more efforts are needed to inform the role.

Second, this study found a positive relationship between SEBs and attitude, which suggested that restaurant companies should enable consumers to express their concerns about their environment through visiting EIRs. Thus, EIRs should emphasize that consumers can participate in environmental protection campaigns by using their restaurants. In fact, a lot of restaurant companies are currently conducting an environmental protection campaign. For instance, Kentucky Fried Chicken (KFC) emphasizes that their business is sustainable by building LEED (Leadership in Energy and Environmental Design)-certified buildings, which leads to about 30 percent savings in energy and water use [86]. Subway is also promoting that they are ecofriendly by emphasizing that only vegetables supplied by farms that use ecofriendly measures are used as ingredients. Thus, if EIRs also emphasize through campaigns that such EIs are produced through environment-friendly measures, consumers will perceive that they can express their environmental concern with EIRs. Consequently, consumers will have a favorable attitude towards visiting EIRs.

Third, the current study found the effect of NEs on attitude in the context of EIRs. In other words, consumers think that they can experience nature through EIRs. It is recommended to produce advertisements that emphasize natural experiences. In fact, green advertising is known to have a critical impact on the consumer decision-making process [87,88]. Therefore, if EIRs appeal to consumers by creating an advertisement against the background of natural environment including forests and mountains, the consumers will recognize that they can experience nature with EIRs. In addition, it is recommended that the physical environment of EIRs creates a natural atmosphere so that customers can have NEs when they eat. As a result of such efforts, consumers are more likely to have a positive attitude toward using the restaurants.

## 5. Conclusions

The purpose of this research was to identify the environmentally friendly role of EIRs, focusing on the concept of PBs. More specifically, this study proposed a theoretical model with eight hypotheses based on the relationships among PBs, attitude, desire, and behavioral intentions. In order to test the proposed hypotheses, 419 responses were collected in Korea. The results of data analysis revealed that the three subdimensions of PBs, i.e., WG, SEBs, and NEs, help to increase attitude. In addition, attitude positively affected desire, which in turn had a positive influence on intentions to use and willingness to pay more. 

## 6. Limitations and Future Research

Although this paper provides meaningful theoretical and managerial implications discussed above, the paper has the following limitations as well. First, the representativeness of the results of this study is an issue because the data for this paper were collected only in Korea. In particular, insect restaurants have not been activated in Korea, so future research is required to collect data from other countries where the restaurants are activated. Second, food culture varies regionally and internationally [89,90]. Thus, it is also meaningful to conduct comparative research on consumers in areas where EIRs are activated and areas where they are not. Third, this study collected data using an online survey, which leads to selection biases [91]; therefore, future research is necessary to employ different types of data collection methods. Fourth, the response rate of 6.94% in this study is somewhat low, so future research needs to consider different kinds of data collection methods in order to enhance response rate. Lastly, 419 samples were employed to assess eight hypotheses in the current study. Although Hair et al. [80] and Weston and Gore [92] suggested that a sample size of 200 is reasonable for performing CFA/SEM, future research is required to collect more data.

## Figures and Tables

**Table 1:** Profile of survey respondents (*n* = 419).

Variable	*n*	Percentage
Gender	211	50.4
Male	208	49.6
Female		
Monthly household income		
6001$ US and over	14	3.3
5001$ US–6000$ US	22	5.3
4001$ US–5000$ US	42	10.0
3001$ US–4000$ US	57	13.6
2001$ US–3000$ US	116	27.7
1001$ US–2000$ US	122	29.1
Under 1000$ US	46	11.0
Marital status		
Single	196	46.8
Married	218	52.0
Widowed/Divorced	5	1.2
Education level		
Less than High school diploma	61	14.6
Associate’s degree	78	18.6
Bachelor’s degree	228	54.4
Graduate degree	52	12.4
Mean age = 37.95 years old		

**Table 2:** Confirmatory factor analysis: items and loadings.

Construct and Scale Items	Standardized Loading ^a^
Psychological benefits Warm glow	
With edible insect restaurants, I can feel good because the restaurant helps to protect the environment.	0.871
With edible insect restaurants, I have the feeling of contributing to the well-being of humanity and nature.	0.909
With edible insect restaurants, I can feel better because the restaurant doesn’t harm the environment.	0.937
Self-expressive benefits	
With edible insect restaurants, I can express my environmental concern.	0.907
With edible insect restaurants, I can demonstrate to myself and my friends that I care about environmental conservation.	0.941
With edible insect restaurants, I can demonstrate to myself and my friends that I care about environmental conservation.	0.928
Nature experiences	
Edible insect restaurants can make me feel close to nature.	0.903
Edible insect restaurants can make me think of nature, fields, forests and mountains.	0.941
Edible insect restaurants can evoke the sensation of being in nature.	0.935
Attitude	
Unfavorable–favorable	0.915
Bad–good	0.899
Negative–positive	0.944
Desires	
I desire to use edible insect restaurants.	0.930
My desire of using edible insect restaurants is strong.	0.972
I want to use edible insect restaurants.	0.963
Intentions to use	
I will dine out at edible insect restaurants.	0.954
I am willing to dine out at edible insect restaurants.	0.958
I am likely to dine out at edible insect restaurants.	0.964
Willingness to pay more	
I am likely to pay more for dining out at edible insect restaurants.	0.945
It is acceptable to pay more for dining out at edible insect restaurants.	0.969
I am likely to spend extra in order to dine out at edible insect restaurants.	0.965
Goodness-of-fit statistics: χ^2^ = 502.646, df = 168, χ^2^/df = 2.992, *p* < 0.001, NFI = 0.961, CFI = 0.974, TLI = 0.967, and RMSEA = 0.069	

Notes 1: ^a^ All factors loadings are significant at *p* < 0.001. Notes 2: NFI = normed fit index, CFI = comparative fit index, TLI = Tucker–Lewis index, and RMSEA = root mean square error of approximation.

**Table 3:** Descriptive statistics and associated measures.

	No. of Items	Mean (SD)	AVE	(1)	(2)	(3)	(4)	(5)	(6)	(7)
(1) Warm glow	3	4.11 (1.24)	0.821	0.932 ^a^	0.806 ^b^	0.702	0.703	0.714	0.707	0.589
(2) Self-expressive benefits	3	3.79 (1.26)	0.856	0.650 ^c^	0.947	0.775	0.672	0.662	0.657	0.605
(3) Nature experiences	3	3.59 (1.35)	0.858	0.493	0.601	0.948	0.622	0.632	0.637	0.314
(4) Attitude	3	4.02 (1.46)	0.846	0.494	0.452	0.387	0.943	0.823	0.829	0.703
(5) Desire	3	3.63 (1.38)	0.912	0.510	0.438	0.399	0.677	0.969	0.851	0.789
(6) Intentions to use	3	3.64 (1.39)	0.919	0.500	0.432	0.406	0.687	0.724	0.971	0.824
(7) Willingness to pay more	3	3.19 (1.28)	0.921	0.347	0.366	0.402	0.494	0.623	0.679	0.972

Notes 1: SD = standard deviation and AVE = average variance extracted. Notes 2: a. Composite reliabilities are along the diagonal. b. Correlations are above the diagonal. c. Squared correlations are below the diagonal.

**Table 4:** Standardized parameter estimates for the structural model.

			Standardized Estimate	*t*-Value	Hypothesis
H1 Warm glow	🡪	Attitude	0.435	6.337 *	Supported
H2 Self-expressive benefits	🡪	Attitude	0.193	3.079 *	Supported
H3 Nature experiences	🡪	Attitude	0.190	2.484 *	Supported
H4 Attitude	🡪	Desire	0.839	22.541*	Supported
H5 Attitude	🡪	Intentions to use	0.152	3.976 *	Supported
H6 Attitude	🡪	Willingness to pay more	0.172	2.669 *	Supported
H7 Desire	🡪	Intentions to use	0.827	19.838 *	Supported
H8 Desire	🡪	Willingness to pay more	0.657	10.125 *	Supported
Goodness-of-fit statistics: χ^2^ = 608.395, df = 178, χ^2^/df = 3.418, *p* < 0.001, NFI = 0.953, CFI = 0.966, TLI = 0.960, and RMSEA = 0.076					

Notes 1: * *p* < 0.05. Notes 2: NFI = normed fit index, CFI = comparative fit index, TLI = Tucker–Lewis index, and RMSEA = root mean square error of approximation.

## Data Availability

Data sharing not applicable.
